# Associations between nutritional status and jumping performance in pre-school children

**DOI:** 10.1371/journal.pone.0333500

**Published:** 2025-09-26

**Authors:** Sanja Ljubičić, Vilko Petrić, Ljubomir Antekolović

**Affiliations:** 1 Faculty of Teacher Education, University of Rijeka; 2 Faculty of Kinesiology, University of Zagreb; Universidad Pablo de Olavide, SPAIN

## Abstract

Although it has been confirmed that overweight and obesity may have negative impact on jumping performance in school-aged children and adolescents, little evidence has been provided for pre-school children. The findings have provided inconclusive results, but mostly that overweight and obese pre-school children do not have an impaired jumping performance, in comparison to their normal weight peers. Therefore, the main purpose of the study was to examine the differences between normal weight and overweight/obese pre-school children in jumping performance outcomes and their correlations with anthropometric indices. Four-hundred and eleven pre-school children with the mean (SD) age = 4.9 (1.1) years, height = 111.2 (9.3) cm, weight = 20.0 (4.2) kg, 53.5% girls were recruited from 34 kindergartens in four major cities. Anthropometric indices included body mass index (BMI), waist-to-hip ratio (WHR), and waist-to-height ratio (WHtR). Normal weight, overweight and obesity were classified according to international norms. Jumping performance was evaluated by the countermovement jump (CMJ) without and with arm swing with Optojump system. In general, pre-school children with overweight and obesity exhibited poorer performance in vertical jumping; they had shorter flight time and flight height, lower power relative to body mass and lower relative strength index (RSI), compared to normal weight children. The strongest correlations were observed between WHtR and jumping outcomes, where higher WHtR values led to shorter flight time and flight height, and lower power relative to body mass in both boys and girls. This study adds new information related to nutritional status and jumping performance in pre-school children. Thus, overweight and obesity need to be considered, when monitoring vertical jumping performance.

## Introduction

Overweight and obesity have become a major public health concern worldwide [1]. Although the prevalence of overweight and obesity has been extremely heterogenous across different geographical regions [2–4], estimates suggest that nearly 15% and 9% of children and adolescents live with overweight and obesity [1]. The existing data show a growing trend [5], and some prediction models suggest that the prevalence of overweight and obesity may double till the year 2050 [6]. Being overweight and/or obese comes from a multicomponent genetic, behavioral and environmental factors [7]. Among many, an unbalanced diet and time spent in sedentary behaviors have been the main causes of overweight and obesity in youth [7–9]. Since being overweight and obese can be observed as a non-communicable disease, evidence shows adverse effects towards cardiovascular, and metabolic health, and the development of a variety of cancers [5]. Unfortunately, the persistence of overweight and obesity during childhood and adolescence periods has been consistently shown to track well into adulthood [10]. Thus, the negative outcomes of being overweight and obese at a young age are two-fold: i) directly impacting health in childhood, and ii) developing chronic disorders and diseases earlier in later life [11,12].

The majority of previous studies have determined the existence of overweight and obesity in children and adolescents ≥5 years old [1,5], while the data regarding early childhood are scarce [13–15]. A region-specific prevalence of overweight and obesity in pre-school children is around 22% in Europe, which greatly exceeds those in Asia (6%), Latin America and Caribbean (7%), Africa (10.4%) and North America (12.1%) [14]. Similar to risk factors for overweight and obesity in older youth [7–9], the most potent are related to child-parent food practices and parental body-mass index [16]. As such, implementing interventions during early childhood should be a cornerstone for managing overweight and obesity during later developmental stages [17].

Vertical jumping is characterized by a stretch-shortening cycle movement, where stretching is referred to as a preparatory period and shortening as a rapid take-off phase during the jump [18]. Based on the pyramid of motor development [19], vertical jumping performance is an essential part of advanced motor skills. Regardless of nutritional status and during the pre-school period, children between ages 3 and 6 years old need to have a strong ability to master basic motor skills, since poor adoption of these movements at an early age may be harmful to execute more complex motor skills in adulthood [20]. Of available literature, most of previous studies have focused on neuromusculoskeletal and biomechanical characteristics of the vertical jumping performance [21,22]. Pre-school children have less joint movement, especially in the knee and hip regions, reducing the power outcome during the take off phase and the utility rate during landing [23].

It has been documented that children with overweight or obesity, opposed to their normal weight peers, have locomotor skills at lower levels [24], including vertical jumping performance [25–27]. However, most data on the relationship between overweight and obesity and jumping performance has been observed in school-going children ≥7 years old [25,28–30], while little evidence has shown a link between excess body weight and jumping performance in pre-school children [31,32]. Opposed to available evidence conducted in children and adolescents [24], most findings in preschoolers did not support the hypothesis, that being overweight or obese would lead to poor motor performance [31,32], making the relationship between excess weight and motor skill development at early ages inconclusive. In a study by Castetbon & Andreyeva [33], obese girls were found to jump shorter than their normal weight peers, while other gross and fine motor skills were not correlated with obesity. The discrepancy may also be attributed to different measures of body composition and implemented motor performance tests to assess jumping performance [24,32].

This study is based on the use of multiple anthropometric indices (BMI, waist circumference, waist-to-height ratio – WHtR, etc.), allowing for a more comprehensive analysis of the relationship between body composition and jumping performance. This approach provides a deeper understanding of the impact of nutritional status on motor development in preschool children, with clear implications for educational and training practice. Therefore, the main purpose of the study was to examine the association between overweight and obesity using the International standard norms [34] and jumping performance in a sample of pre-school children aged 3–6 years. Although we could not make a conclusive assumption about the nature of the association [31–33], we hypothesized that overweight and obese children would exhibit poorer values in jumping performance, in comparison to their normal weight peers.

## Materials and methods

### Study participants

In this observational, cross-sectional study, we recruited 411 pre-school boys and girls; mean (SD) [age = 4.9 (1.1) years, height = 111.2 (9.3) cm, weight = 20.0 (4.2) kg, 53.5% girls] selected from 34 kindergartens in Rijeka (Croatia), Zagreb (Croatia), Ljubljana (Slovenia) and Koper (Slovenia). The inclusion criteria involved children aged 3−6 years with typical development and without any locomotor or mental disorders and diseases, who were enrolled in day care. Before entering the study, parents or guardians were instructed about the main aims and hypotheses of the study, the dissemination of the findings and potential benefits for their children. Each child’s parents/guardians provided written consent before data collection. The research related to human use has been complied with all the relevant national regulations, institutional policies and in accordance the tenets of the Helsinki Declaration [35] and has been approved by the Faculty of Teacher Education, University of Rijeka (Ethical approval no. uniri-mladi-drustv-23–37: KLASA:641–01/24–01/03; URBROJ: 2170-1-38-10-24-2; uniri-iskusni-drustv-23–201: KLASA: 641–01/24–01/03; URBROJ: 2170-1-38-10-24-1). The study was conducted from May 17 to November 19, 2024.

### Jumping performance

To assess jumping performance, we used the reliable and valid Optojump photocell system (Microgate, Bolzano, Italy), an infrared platform consisted of two parallel bars (receiver and transmitter units), which were placed 1 meter apart in a parallel position to each other. Both bars were connected to a personal computer with software to quantify jump height [36]. The flight time of vertical jumps was measured with an accuracy of 1/1000 seconds and the estimated jump height was calculated by the following formula: 9.81 x flight time^2^/8 [37]. In order to examine jumping performance, we selected two tests: countermovement jump (CMJ) without arm swing and CMJ with arm swing [36,38]. Both tests were equally performed, starting from the fully upright position with knees fully extended and the feet shoulder-width apart. Children were instructed to execute a fast downward movement with approximately 90° knee flexion and a fast upward movement to jump as high as possible, by keeping the hands on the hips throughout the whole movement (first test), or by swinging back with the arms during the downward movement and forward during the upright movement [38]. Each test was performed 5 times with a 5-min rest period between each trial. Standardized verbal instructions and demonstrations were provided before testing to ensure consistency across participants and test administrators. The CMJ tests have been extensively used to assess vertical performance and power output, and their reliability, validity and utility properties have been confirmed in children [38]. The Optojump software generated data regarding contact time (s), flight time (s), jump height (cm), power (W/kg), pace (steps/s), reactive strength index (RSI; m/s) and verticality [39].

### Anthropometric indices

The classification of overweight/obesity status was assessed by several well-established anthropometric indices, including body mass index (BMI), waist circumference (WC), hip circumference (HC), waist-to-hip ratio (WHR) and waist-to-height ratio (WHtR) [40,41]. Height and weight were objectively measured using stadiometer and digital scale with a precision of 0.1 cm and 0.1 kg. BMI was calculated by the following formula: [weight (kg)/height(m)^2^]. Overweight (the 85^th^ percentile) and obesity (the 95^th^ percentile) were defined for sex and age, according to previous studies [34]. WC was measured with anthropometric tape placed horizontally midway between the lower rib margin and the iliac crest at the end of normal expiration while standing still, while HC was measured at the largest circumference around the buttocks [41]. Finally, WHR and WHtR were obtained by dividing WC with HC and WC with height (in cm). WHT and WHtR were used as indicators of abdominal fat, with values above 85^th^ percentile and a cut off of 0.5 to identify abdominal obesity in children [42,43].

### Data analysis

The Kolmogorov-Smirnov test was used to assess the level of data normality. Means and standard deviations (SD) were used to present the data for normally distributed variables and medians with interquartile range (25^th^ - 75^th^ percentile) for not normally distributed variables. Differences between ‘normal weight’ and ‘overweight/obese’ pre-school children in vertical jumping outcomes were examined using Student t-test for independent samples or Man-Whitney U-test. Pearson’s and Spearman’s coefficients of correlation were used to examine the relationship between anthropometric indices and each of the vertical jumping outcome. Two-sided *p*-values were used, and significance was set at α < 0.05. All the analyses were calculated in Statistical Packages for Social Sciences v.23 (SPSS, Chicago, IL, United States).

## Results

Table 1 exhibits sex- and age-specific data for various anthropometric indices. Results showed no significant ‘sex’ differences, indicating that values were similar for both boys and girls, respectively (p > 0.05). However, the factor ‘age’ significantly influenced the results, where older boys and girls had higher values in BMI, WC, and HC, opposed to their younger counterparts (p < 0.05). For WHR and WHtR, the data suggested linear decreases by age, where younger boys and girls exhibited higher values in given parameters, compared to their older peers (p < 0.05).

**Table 1:** Characteristics of the study participants, according to sex and age, data are presented as mean (SD).

Sex	Age (yrs)	Height (cm)	Weight (kg)	BMI (kg/m^2^)	WC (cm)	HC (cm)	WHR	WHtR
Boys	3 (N = 43)	101.0 (5.1)	16.9 (2.2)	16.3 (1.3)	53.7 (3.1)	58.7 (3.2)	0.92 (0.05)	0.53 (0.04)
	4 (N = 62)	108.4 (4.9)	18.6 (2.0)	15.6 (1.0)	54.0 (3.3)	60.6 (3.2)	0.89 (0.05)	0.50 (0.03)
	5 (N = 42)	116.4 (4.6)	21.4 (3.0)	15.6 (1.5)	54.6 (3.7)	62.8 (4.5)	0.87 (0.05)	0.47 (0.03)
	6 (N = 39)	123.4 (5.4)	25.1 (4.5)	16.2 (2.0)	56.8 (4.7)	66.5 (6.6)	0.86 (0.07)	0.46 (0.03)
	Total (N = 186)	111.7 (9.4)	20.2 (4.2)	15.9 (1.4)	54.6 (3.8)	61.9 (5.2)	0.89 (0.06)	0.49 (0.04)
Girls	3 (N = 62)	101.0 (4.6)	16.2 (1.7)	15.7 (1.2)	53.0 (3.3)	57.3 (3.2)	0.93 (0.05)	0.53 (0.04)
	4 (N = 61)	109.3 (5.0)	18.8 (2.4)	15.5 (1.3)	53.8 (4.1)	60.7 (4.0)	0.89 (0.05)	0.49 (0.04)
	5 (N = 55)	114.9 (5.2)	21.2 (3.4)	15.8 (1.8)	53.9 (4.1)	63.5 (4.8)	0.85 (0.05)	0.47 (0.04)
	6 (N = 41)	122.6 (5.9)	24.5 (4.5)	16.1 (2.1)	56.3 (5.5)	66.4 (5.4)	0.85 (0.04)	0.46 (0.04)
	Total (N = 219)	110.8 (9.2)	19.8 (4.2)	15.8 (1.6)	54.1 (4.3)	61.5 (5.4)	0.88 (0.06)	0.49 (0.05)

**Abbreviations:** BMI (Body mass index), WC (waist circumference), HC (hip circumference), WHT (waist-to-hip ratio), WHtR (waist-to-height ratio); p < 0.05

Differences in various anthropometric indices observed for CMJ without arm swing in boys and girls are presented in Table 2. In boys, children with ‘normal’ BMI had significantly higher values in power, pace and RSI, compared to ‘overweight/obese’ children (p < 0.05). No significant differences in other CMJ outcomes without arm swing were observed (p > 0.05). In girls, no significant differences in those with ‘normal’ vs ‘overweight/obese’ were shown, indicating that both groups had similar values (p > 0.05). When observing differences between ‘normal’ vs. ‘overweight/obese’ children according to WHR, results showed no significant differences between the two groups in boys. In girls, those with ‘normal’ weight had significantly higher values in flight, height, power, RSI and verticality, opposed to their ‘overweight/obese’ counterparts (p < 0.05). Finally, when examining differences between ‘normal’ vs. ‘overweight/obese’ boys according to WHtR, boys with ‘normal’ weight exhibited shorter contact time, longer flight time and flight height, larger power, pace, RSI and verticality (p < 0.05). In girls, those with ‘normal’ weight exhibited longer flight time and flight height, larger power and RSI values, opposed to ‘overweight/obese’ peers (p < 0.05).

**Table 2:** Differences between normal vs. overweight/obese children in various anthropometric indices in CMJ without arm swing; data are presented as mean (SD) or median (IQR).

Sex	Measure	BMI		WHR		WHtR	
		Normal	OW/OB	Normal	OW/OB	Normal	OW/OB
Boys	Contact time (s)	1.4 (0.9)	1.7 (1.1)	1.4 (1.0)	1.6 (0.6)	1.3 (0.8)	2.0 (1.5)**
	Flight time (s)	0.3 (0.05)	0.3 (0.05)	0.3 (0.05)	0.3 (0.06)	0.30 (0.05)	0.26 (0.05)***
	Height (cm)	11.3 (3.9)	11.1 (3.3)	11.3 (3.7)	11.3 (4.8)	11.7 (3.7)	8.9 (3.2)***
	Power (W/kg)	9.6 (3.3)	8.4 (2.2)*	9.5 (3.1)	8.2 (3.5)	9.7 (3.1)	7.4 (2.6)***
	Pace (steps/s)	0.8 (0.5 - 1.4)	0.6 (0.4 - 0.9)*	0.8 (0.5 - 1.3)	0.6 (0.6 - 0.8)	0.8 (0.5 - 1.3)	0.6 (0.4 - 0.8)*
	RSI (m/s)	0.10 (0.06 - 0.20)	0.07 (0.04 - 0.15)*	0.10 (0.06 - 0.19)	0.06 (0.04 - 0.09)	0.10 (0.06 - 0.20)	0.06 (0.03 - 0.10)*
	Verticality	2.4 (1.3 - 4.9)	2.5 (0.9 - 6.1)	2.5 (1.3 - 4.9)	1.6 (0.8 - 5.9)	2.7 (1.4 - 5.3)	1.4 (0.9 - 3.2)***
Girls	Contact time (s)	1.3 (1.0)	1.4 (0.8)	1.3 (1.0)	1.5 (0.9)	1.3 (1.1)	1.4 (0.8)
	Flight time (s)	0.3 (0.05)	0.29 (0.05)	0.30 (0.05)	0.26 (0.06)***	0.31 (0.05)	0.27 (0.04)***
	Height (cm)	11.4 (3.6)	10.4 (3.4)	11.6 (3.4)	8.7 (3.7)***	11.7 (3.5)	8.9 (2.9)***
	Power (W/kg)	9.6 (3.6)	8.9 (2.5)	9.8 (3.4)	7.7 (3.4)**	9.8 (3.6)	8.1 (2.3)**
	Pace (steps/s)	0.7 (0.5 - 1.0)	0.6 (0.5 - 1.1)	0.7 (0.5 - 1.1)	0.7 (0.5 - 0.9)	0.7 (0.5 - 1.0)	0.7 (0.5 - 1.1)
	RSI (m/s)	0.09 (0.06 - 0.20)	0.08 (0.05 - 0.20)	0.09 (0.06 - 0.18)	0.06 (0.04 - 0.08)*	0.09 (0.06 - 0.18)	0.08 (0.04 - 0.16)
	Verticality	2.4 (1.5 - 4.2)	2.1 (1.0 - 3.0)	2.4 (1.5 - 4.2)	1.8 (1.1 - 3.6)*	2.4 (1.5 - 4.3)	1.7 (1.1 - 2.7)*

**Abbreviations:** BMI (Body mass index), WHR (waist-to-hip ratio), WHtR (waist-to-height ratio), RSI (reactive strength index); ***p < 0.001; **p < 0.01; *p < 0.05

Correlations between CMJ without arm swing and various anthropometric indices are presented in Table 3. BMI was not significantly correlated with CMJ outcomes in boys and girls. For WHR, negative correlations with flight time, height and power were observed for both boys and girls, indicating that higher values in WHR denoted lower flight time, height and power. In girls, WHR was also correlated with lower RSI and verticality. In boys, WHtR was significantly positively correlated with contact time, while negative correlations with flight time, height, power, pace and RSI were observed. For girls, similar correlations were shown, except for contact time and pace, where there was no significant correlation with WHtR, but a negative correlation with verticality existed.

**Table 3:** Correlations between anthropometric indices and outcomes of CMJ without arm swing in preschool children.

Sex	Measure	BMI	WHR	WHtR
Boys	Contact time (s)	0.13^a^	0.13^a^	0.25^a^ ***
	Flight time (s)	0.01^a^	−0.27^a^ ***	−0.40^a^ ***
	Height (cm)	−0.01^a^	−0.25^a^ ***	−0.41^a^ ***
	Power (W/kg)	−0.06^a^	−0.21^a^ *	−0.34^a^ ***
	Pace (steps/s)	−0.06^b^	0.04^b^	−0.20^b^ *
	RSI (m/s)	−0.06^b^	−0.15^b^ *	−0.23^b^ *
	Verticality	−0.08^b^	−0.06^b^	−0.11^b^
Girls	Contact time (s)	−0.09^a^	0.07^a^	0.05^a^
	Flight time (s)	−0.11^a^	−0.43^a^ ***	−0.54^a^ ***
	Height (cm)	−0.11^a^	−0.42^a^ ***	−0.52^a^ ***
	Power (W/kg)	−0.04^a^	−0.28^a^ ***	−0.33^a^ ***
	Pace (steps/s)	0.05^b^	0.01^b^	−0.01^b^
	RSI (m/s)	−0.02^b^	−0.19^b^ **	−0.24^b^ ***
	Verticality	−0.05^b^	−0.14^b^ *	−0.15^b^ *

**Abbreviations:** BMI (Body mass index), WHR (waist-to-hip ratio), WHtR (waist-to-height ratio), BAI (body adiposity index); *p < 0.05; **p < 0.01; ***p < 0.001; ^a^denotes using Pearson’s product – moment correlation coefficients; ^b^denotes using Spearman’s rank – order correlation coefficients.

Differences in various anthropometric indices observed for CMJ with arm swing in boys and girls are presented in Table 4. In boys, children with ‘normal’ BMI had significantly lower values in contact time and higher values in RSI, compared to ‘overweight/obese’ children (p < 0.05). No significant differences in other CMJ outcomes without arm swing were observed (p > 0.05). In girls, no significant differences in those with ‘normal’ vs ‘overweight/obese’ were shown, except for power and RSI, where those with ‘normal’ weight exhibited larger values, opposed to ‘overweight/obese’ counterparts. When observing differences between ‘normal’ vs. ‘overweight/obese’ children according to WHR, results showed no significant differences between the two groups in boys. In girls, those with ‘normal’ weight had significantly lower contact time, longer flight time, height, power, pace and RSI, opposed to their ‘overweight/obese’ counterparts (p < 0.05). Finally, when examining differences between ‘normal’ vs. ‘overweight/obese’ boys according to WHtR, boys with ‘normal’ weight exhibited shorter contact time, longer flight time and flight height, larger power, pace, RSI and verticality (p < 0.05). In girls, those with ‘normal’ weight exhibited longer flight time and flight height, larger power, RSI and verticality values, opposed to ‘overweight/obese’ peers (p < 0.05).

**Table 4:** Differences between normal vs. overweight/obese children in various anthropometric indices in CMJ with arm swing; data are presented as mean (SD) or median (IQR).

Sex	Measure	BMI		WHR		WHtR	
		Normal	OW/OB	Normal	OW/OB	Normal	OW/OB
Boys	Contact time (s)	1.7 (1.0)	2.31 (1.04)**	1.7 (1.2)	1.9 (1.5)	1.6 (1.0)	2.4 (2.1)**
	Flight time (s)	0.32 (0.06)	0.32 (0.04)	0.32 (0.05)	0.33 (0.06)	0.33 (0.05)	0.28 (0.04)***
	Height (cm)	13.0 (4.5)	12.7 (3.0)	12.9 (4.2)	13.7 (5.8)	13.4 (4.3)	10.0 (2.7)***
	Power (W/kg)	9.4 (3.0)	8.6 (1.7)	9.2 (2.8)	9.5 (2.6)	9.5 (2.9)	7.4 (1.3)***
	Pace (steps/s)	0.59 (0.47 - 0.82)	0.47 (0.34 - 0.61)	0.58 (0.46 - 0.79)	0.47 (0.31 - 0.84)	0.59 (0.41 - 0.87)	0.41 (0.31 - 0.61)*
	RSI (m/s)	0.08 (0.05 - 0.12)	0.06 (0.04 - 0.09)***	0.08 (0.05 - 0.12)	0.06 (0.05 - 0.15)	0.09 (0.05 - 0.12)	0.05 (0.03 - 0.06)*
	Verticality	1.9 (1.0 - 3.7)	1.7 (0.9 - 5.6)	1.9 (1.0 - 4.0)	1.7 (1.1 - 2.2)	1.9 (1.0 - 4.5)	1.8 (1.1 - 2.7)**
Girls	Contact time (s)	1.64 (0.9)	1.6 (0.7)	1.6 (0.8)	2.0 (1.3)*	1.7 (0.9)	1.5 (0.7)
	Flight time (s)	0.32 (0.06)	0.30 (0.06)	0.32 (0.06)	0.27 (0.05)***	0.32 (0.06)	0.29 (0.05)***
	Height (cm)	12.8 (4.4)	11.1 (4.3)	13.0 (4.3)	9.3 (3.2)***	13.0 (4.4)	10.3 (3.7)***
	Power (W/kg)	9.3 (2.7)	8.2 (2.1)*	9.4 (2.7)	7.5 (2.2)***	9.4 (2.7)	7.9 (2.1)***
	Pace (steps/s)	0.61 (0.49 - 0.84)	0.58 (0.46 - 0.77)	0.61 (0.51 - 0.82)	0.52 (0.43 - 0.79)*	0.60 (0.49 - 0.81)	0.62 (0.47 - 0.82)
	RSI (m/s)	0.09 (0.05 - 0.12)	0.07 (0.04 - 0.09)*	0.09 (0.05 - 0.12)	0.06 (0.03 - 0.08)*	0.09 (0.05 - 0.12)	0.06 (0.04 - 0.09)**
	Verticality	1.8 (0.8 - 3.5)	2.0 (1.1 - 4.0)	1.9 (0.9 - 3.7)	1.8 (1.1 - 3.7)	1.9 (1.0 - 3.9)	1.6 (0.8 - 3.5)***

**Abbreviations:** BMI (Body mass index), WHR (waist-to-hip ratio), WHtR (waist-to-height ratio), RSI (reactive strength index); ***p < 0.001; **p < 0.01; *p < 0.05

Correlations between CMJ with arm swing and various anthropometric indices are presented in Table 5. BMI was only significantly correlated with higher contact time in boys, while no observed significant correlations in girls were shown. For WHR, negative correlations with flight time, height and power were observed for both boys and girls, indicating that higher values in WHR denoted lower flight time, height and power. In girls, WHR was also correlated with lower RSI. In boys, WHtR was significantly positively correlated with contact time, while negative correlations with flight time, height, power, RSI and verticality were observed. For girls, similar correlations were shown, except for contact time, where there was no significant correlation with WHtR.

**Table 5:** Correlations between anthropometric indices and outcomes of CMJ with arm swing in preschool children.

Sex	Measure	BMI	WHR	WHtR
Boys	Contact time (s)	0.21^a^**	0.10^a^	0.20^a^ **
	Flight time (s)	−0.05^a^	−0.23^a^ ***	−0.48^a^ ***
	Height (cm)	−0.06^a^	−0.21^a^ **	−0.48^a^ ***
	Power (W/kg)	−0.09^a^	−0.20^a^ **	−0.40^a^ ***
	Pace (steps/s)	−0.09^b^	−0.02^b^	−0.06^b^
	RSI (m/s)	−0.11^b^	−0.11^b^	−0.24^b^ **
	Verticality	0.01^b^	−0.12^b^	−0.16^b^ *
Girls	Contact time (s)	−0.10^a^	0.16^a^ *	0.11^a^
	Flight time (s)	−0.10^a^	−0.43^a^ ***	−0.54^a^ ***
	Height (cm)	−0.09^a^	−0.45^a^ ***	−0.55^a^ ***
	Power (W/kg)	−0.07^a^	−0.38^a^ ***	−0.48^a^ ***
	Pace (steps/s)	0.01^b^	−0.11^b^	−0.13^b^
	RSI (m/s)	−0.06^b^	−0.22^b^ ***	−0.31^b^ ***
	Verticality	0.01^b^	−0.13^b^	−0.18^b^ **

**Abbreviations:** BMI (Body mass index), WHR (waist-to-hip ratio), WHtR (waist-to-height ratio); *p < 0.05; **p < 0.01; ***p < 0.001; ^a^denotes using Pearson’s product – moment correlation coefficients; ^b^denotes using Spearman’s rank – order correlation coefficients.

## Discussion

The main purpose of the study was to examine the association between overweight and obesity and jumping performance in a sample of pre-school children aged 3–6 years. Findings of this study suggest, that ‘overweight/obese’ pre-school children exhibit poorer performance in jumping, compared to their ‘normal weight’ peers. The strongest differences between ‘normal weight’ vs. ‘overweight/obese’ pre-school children are observed, when WHtR is used as an indicator. Also, WHtR is moderately correlated with flight, height and power in both boys and girls, compared to other anthropometric indices.

Previous studies have predominantly used BMI, as an indicator for overweight and obesity and found no significant differences between ‘normal weight’ and ‘overweight/obese’ pre-school children [31–33]. Specifically, a study by Nervik *et al*. [31] showed that BMI and gross motor performance in pre-school children aged 3–5 years were not significantly correlated. Similar observations were found in a study by Wood *et al*. [32], a set of regression analyses indicated that the fat mass percentage nor sum of skinfolds was correlated with locomotor skills in 342 pre-school children. Another study only found that BMI z-score was negatively correlated with passing the hop test in boys and girls, yet no significant differences in other gross or fine locomotor skills were observed [33]. However, some studies showed that obese girls only exhibited poorer jumping ability, compared to their ‘normal weight’ peers [33,44]. However, the problem of using BMI as an indicator for overweight and obesity is two-fold. First, the BMI specificity to define overweight (the 85^th^ percentile) and obesity (the 95^th^ percentile) could lead to a misclassification of boys and girls, who were more muscular and physically active. Indeed, we used the US Centers for Disease Control and Prevention Growth normative values to classify children into ‘normal weight’ and ‘overweight/obesity’ [34], and those values seemed to be somewhat higher than the International Obesity Task Force [45]. This would imply that higher BMI values might be more attributable to muscle, rather than the fat mass, leading to lower rates of misclassification of muscular boys and girls at lower age [33]. On the other hand, the classification of boys and girls according to WHR and WHtR seemed to exhibit clearer differences and correlations with vertical jumping outcomes. This is not surprising, since previous evidence has suggested that WHT [43] and WHtR [46] are more strongly correlated with objective measures of fat mass percentage, opposed to BMI. The disadvantage of BMI lies in its inability to distinguish between lean muscle mass and fat mass and may not be suitable as a measure for overweight and obesity in pre-school children, because of their muscular development and body weight status [33].

From a biomechanical point of view, overweight children monitored with a motion capture to establish kinematic and kinetic differences with ‘normal weight’ were shown to exhibit lower jumping performance relative to their mass, shallower countermovement range of motion in the knee and hip regions, lower hip torque and hip work, and earlier peak joint angular velocities [26], which was confirmed by a recent systematic review and meta-analysis [27]. Physiologically, jumping movements require a relatively high amount of energy expenditure, which can potentially be more exhausting for overweight and obese children, discouraging them to participate in jumping-related activities and accumulating excessive fat [47]. This is in line with our findings, where overweight and obese boys and girls (obtained from WHT or WHtR) had shorter flight time and jump height, and lower level of power, irrespective from the type of the test (CMJ without or with arm swing).

Although differences in jumping performances in relation to nutritional status have been inconsistent between the studies [31–33], significant differences in this study could not be fully attributed to overweight or obesity but should also be observed by biological maturation [24]. Changes in gross and fine motor development are not the same as in body size components, and differences between ‘normal weight’ and ‘overweight/obese’ children become more pronounced with older age [24]. Thus, biological maturation needs to be considered when examining anthropometric and musculoskeletal performance of pre-school children, due to annual changes that display at different rates.

Regardless of previous studies that found no significant differences in jumping performance between ‘normal weight’ and ‘overweight/obese’ pre-school children, this study corroborates the hypothesis that overweight and obesity is correlated with poorer performance, when considering jumping activities. Since the effects of overweight and obesity on motor performance have been confirmed in school-going children and adolescents, but not in pre-school children, this study is one of a few that confirms the existence of differences among pre-school children with different nutritional status in jumping performance.

Our results indicate that jumping ability is not merely a part of natural development but is strongly influenced by a child’s body composition and nutritional status. Current approaches to jump training in children often rely on the assumption that these abilities will develop spontaneously, without the need for targeted interventions. However, our data demonstrate that this is not always the case—children with excess body weight exhibit reduced jumping performance, which may further impact their participation in physical activities and potentially slow their motor development in the long term.

This study is not without limitations. First, due to a cross-sectional design, we cannot establish the causality effect between the anthropometric indices and jumping tests. For example, pre-school children with overweight and obesity need to move greater mass to act in the opposite direction from the center of gravity, directly affecting biomechanical and physiological properties of the body. On the other hand, it has been observed that hopping and jumping activities require more energy expenditure to complete, which may discourage them to participate in such activities and accumulate more fat mass. Second, to classify pre-school children into ‘normal weight’ and ‘overweight/obese’, we used BMI, WHT and WHtR. Although these indices have been widely used in these populations [34,42,43], more objective methods (doubly labelled water, dual-energy X-ray absorptiometry) might provide different results. Third, we did not assess maturation, limiting the explanation of the biological patterns between overweight and obesity and jumping performance.

Fourth, we were not able to measure additional study covariates that could serve as adjusted variables in our analyses, nor to examine physical activity patterns using accelerometers. Future research should take into account biological maturation and children’s sensitivity to measurement instruments, as well as employ more objective methods to assess the nutritional status of pre-school children, in order to overcome these challenges in the future.

## Conclusions

In summary, this study shows that overweight and obese boys and girls have poorer jumping performance, compared to their ‘normal weight’ peers. The differences are mainly observed in shorter flight time, shorter flight height and less power relative to body mass. These findings have significant implications for educational and training practices. In the context of currently prevailing approaches, where the development of jumping abilities is often left to spontaneous maturation, our data suggest the need for more targeted and individualized interventions, especially for children at increased risk of overweight. Jumping, as an important form of explosive movement, requires specific biomechanical and physiological prerequisites that are impaired in obese children, which may further hinder their participation in physical activities and consequently negatively affect their overall development. Therefore, we propose the introduction of structured activities in preschool programs aimed at promoting jumping performance with proper biomechanical technique, particularly for children at higher risk of excess body weight. Additionally, it is important that the application of anthropometric indices in educational and training contexts is not viewed solely through a diagnostic lens but rather as a tool to guide individualized motor development programs.
